# Risk factors for hospital outcomes in pulmonary embolism: A retrospective cohort study

**DOI:** 10.3389/fmed.2023.1120977

**Published:** 2023-04-11

**Authors:** Giorgia Lüthi-Corridori, Stéphanie Giezendanner, Cedrine Kueng, Maria Boesing, Anne B. Leuppi-Taegtmeyer, Munachimso Kizito Mbata, Philipp Schuetz, Joerg D. Leuppi

**Affiliations:** ^1^Cantonal Hospital Baselland, University Center of Internal Medicine, Liestal, Switzerland; ^2^Faculty of Medicine, University of Basel, Basel, Switzerland; ^3^Centre for Primary Health Care, University of Basel, Basel, Switzerland; ^4^Department of Patient Safety, Medical Directorate, University Hospital Basel, Basel, Switzerland; ^5^Cantonal Hospital Aarau, University Department of Medicine, Aarau, Switzerland

**Keywords:** LOHS, pulmonary embolism, risk factors, length of hospital stay, multi morbidity, rehospitalization, in hospital death

## Abstract

**Background:**

Pulmonary embolism (PE) is not only a life-threatening disease but also a public health issue with significant economic burden. The aim of the study was to identify factors—including the role of primary care—that predict length of hospital stay (LOHS), mortality and re-hospitalization within 6 months of patients admitted for PE.

**Method:**

A retrospective cohort study was conducted with patients presenting to a Swiss public hospital with PE diagnosed at the hospital between November 2018 and October 2020. Multivariable logistic and zero-truncated negative binomial regression analyses were performed to assess risk factors for mortality, re-hospitalization and LOHS. Primary care variables encompassed whether patients were sent by their general practitioner (GP) to the emergency department and whether a GP follow-up assessment after discharge was recommended. Further analyzed variables were pulmonary embolism severity index (PESI) score, laboratory values, comorbidities, and medical history.

**Results:**

A total of 248 patients were analyzed (median 73 years and 51.6% females). On average patients were hospitalized for 5 days (IQR 3–8). Altogether, 5.6% of these patients died in hospital, and 1.6% died within 30 days (all-cause mortality), 21.8% were re-hospitalized within 6 months. In addition to high PESI scores, we detected that, patients with an elevated serum troponin, as well as with diabetes had a significantly longer hospital stay. Significant risk factors for mortality were elevated NT-proBNP and PESI scores. Further, high PESI score and LOHS were associated with re-hospitalization within 6 months. PE patients who were sent to the emergency department by their GPs did not show improved outcomes. Follow-up with GPs did not have a significant effect on re-hospitalization.

**Conclusion:**

Defining the factors that are associated with LOHS in patients with PE has clinical implications and may help clinicians to allocate adequate resources in the management of these patients. Serum troponin and diabetes in addition to PESI score might be of prognostic use for LOHS. In this single-center cohort study, PESI score was not only a valid predictive tool for mortality but also for long-term outcomes such as re-hospitalization within 6 months.

## 1. Introduction

Pulmonary embolism (PE) is a serious, potentially life-threatening health condition that represents the third major cause of cardiovascular death behind myocardial infarction and cerebrovascular accidents (1, 2). PE can be considered a central public health issue since it is associated with a substantial economic burden (3–6).

The exact incidence rate for PE is not available but estimates range from 39 to 115 per 100,000 population. Additionally, as the incidence of PE rises with age, PE rates can be expected to continue increasing even further due to the rapidly ageing population in high-income countries, and therefore to significantly impact morbidity, mortality, and healthcare costs (6, 7). Most nations have an urgent dilemma in the realm of public health: how to address the difficulties brought on by the rise in the number of PE patients while utilizing the available medical resources to better fulfill their medical demand without impacting on cost and overtreatments.

Over the past decades, the incidence rate of PE has increased in Europe, whereas the mortality rate and the length of hospital stay (LOHS) have slightly decreased, due to advances in treatments and diagnostics (8–12). In a retrospective, Italian cohort study of 328 patients with PE, despite a trend in reduction in LOHS, the mean and median have not significantly decreased, due to a very small percentage (3%) of patients who received an ultra-early discharge and a large percentage of patients who were discharged within 6 days (31.5%) (13). LOHS is considered a crucial characteristic for health reports when it comes to the management and evaluation of inpatients and is a significant signal for the assessment of hospitals’ service quality (14). Several factors can influence LOHS in patients with PE such as sociodemographic, health-related characteristics and hospital care-related features (15–17). Due to the wide variability of influencing factors, there is no uniform approach to predict the length of stay for PE.

The primary aim of the study was to identify which factors may affect the length of stay of patients admitted for PE. The identification of patient characteristics influencing LOHS may allow decision-makers to plan hospital management accordingly.

Particularly we retrospectively explored if the primary outcome length of hospital stay for PE was influenced by commonly available sociodemographic and health-related variables measurable at entry time.

Although a reduced length of stay decreases hospital costs, it might negatively affect the quality of care. For this reason, as secondary outcomes, we analyzed factors associated with all-cause mortality (in hospital or 30 days mortality) and rehospitalization within 6 months.

## 2. Materials and methods

### 2.1. Design and setting

Our study was conducted in the cantonal hospital of Baselland (KSBL), a district general hospital covering a stable population of 280,000 in Northwest Switzerland. We undertook a retrospective cohort study with 378 consecutive patients hospitalized at the KSBL at the medical or surgical ward and who received the diagnosis of pulmonary embolism (according to the primary International Classification of Disease codes) during their hospital stay between November 2018 and October 2020. We were able to access the electronic case notes of 378 patients to retrieve presenting symptoms and clinical signs that have been associated with PE. Further, socio-demographics, vital signs, comorbidities and discharge variables (mortality and re-hospitalization) were assessed.

### 2.2. Inclusion and exclusion criteria

Data of these patients were individually reviewed. Patients were included in the study if a new PE was the main reason for their hospitalization and their diagnosis was confirmed by computer tomographic pulmonary angiogram (CTPA), scintigraphy or duplex ultra-sound by a specialist (deep vein thrombosis (DVT) combined with PE specific symptoms) within 12 h after presentation. Alternatively, they were also included if confirmatory, diagnostic imaging was performed later, but anticoagulant treatment was started within 12 h after presentation to the hospital due to high clinical suspicion of PE.

The following patients were excluded:

• Denied research consent.

• PE only as a suspected diagnosis and never confirmed with any imaging method.

• Primarily hospitalized for another reason, and PE was diagnosed after >12 h.

• Transferred from/to another hospital and therefore no complete case documentation.

After the application of the eligibility criteria, 248 patients were included in the analysis.

### 2.3. Statistical analyses

The outcome variables comprised LOHS (primary outcome), all-cause mortality in hospital and 30 days, and re-hospitalization within 6 months (secondary outcomes).

To minimize the risk of bias, optimism, and overfitting, no data-driven selection of variables was done. We selected potential predictors based on the literature and on clinical knowledge. Two researchers conducted a literature review and consulted clinical experts in the field. All variables are included in Table 1. Predictors included the PESI score based on age at entry, sex, history of cancer, history of chronic lung disease, history of heart failure, respiratory rate, hypothermia (below 36 degrees Celsius), systolic blood pressure (BP) <100 mmHg, heart rate (HR) ≥110 bpm, O_2_ saturation (SpO_2_) below 90%, altered mental status, and respiratory rate ≥ 30/min (18).

**Table 1:** Patient characteristics.

Demographic	All (*n* = 248)	Missing *n* (%)
Age at diagnosis, median (IQR) in years	73 (62–81.5)	--
Gender (female)	128 (51.6%)	--
Insurance type		
General	199 (80.2%)	--
Half-private	30 (12.1%)	--
Private	19 (7.7%)	--
Vital signs		
Heart rate (bpm), mean (SD)	92.1 (19.7)	--
Tachycardia (>100 bpm)	76 (30.6%)	--
Blood pressure systolic (mmHg), mean (SD)	140.7 (24.9)	--
Blood pressure diastolic (mmHg), mean (SD)	83.1 (14.1)	--
Hypotension (<100/60 mmHg), *n* (%)	11 (4.4%)	--
Hypertension (>140/90 mmHg), *n* (%)	124 (50%)	--
Respiratory rate (/min) mean (SD)	20.4 (5.7)	35 (14.1%)
Hypothermia (<36°C), *n* (%)	9 (3.6%)	--
Oxygen saturation, mean (SD)	93.8 (4.8)	--
Oxygen requirement	36 (14.5%)	--
Comorbidities	229 (92.3%)	--
Dyslipidemia, *n* (%)	44 (17.7%)	--
Diabetes, *n* (%)	37 (14.9%)	--
Cardiovascular disease, *n* (%)	86 (34.7%)	--
Heart failure, *n* (%)	5 (2%)	--
Chronic lung disease, *n* (%)	44 (17.7%)	--
Rheumatic disease, *n* (%)	30 (12.1%)	--
Mental disease, *n* (%)	55 (22.2%)	--
Altered mental status, *n* (%)	5 (2.2%)	--
Active cancer, *n* (%)	30 (12.1%)	--
Medical history		
Previous VTE, *n* (%)	58 (23.4%)	--
Previous PE, *n* (%)	26 (10.5%)	--
Previous DVT, *n* (%)	44 (17.7%)	--
History of cancer, *n* (%)	50 (20.2%)	--
History of hypertension, *n* (%)	126 (50.8%)	--
PESI		
PESI score, mean (SD)	96.8 (31.4)	--
PESI retrospectively calculated, *n* (%)	200 (81%)	
Laboratory values		
NT-proBNP, (ng/L) median (IQR)^*^	499 (125–2,479)	70 (28.2%)
Troponin-T hs (ng/L), median (IQR)^**^	16.95 (7.22–44.38)	94 (37.9%)
Entry and discharge circumstances		
Housing situation before admission		
Private home, *n* (%)	211 (85.1%)	--
Care facility, *n* (%)	37 (14.9%)	--
Sent by GP, *n* (%)	122 (49.2%)	--
Follow-up with GP, *n* (%)	72 (29%)	Data of 14 patients missing (died)
Discharge destination		Data of 14 patients missing (died)
Private home, *n* (%)	200 (80.6%)	
Care facility, *n* (%)	34 (13.7%)	
Rehabilitation unit, *n* (%)	21 (8.5%)	Data of 14 patients missing (died)
Outcomes		
Length of stay, in nights, median (IQR)	5 (3–8)	--
	Median 5 (3–8)	
Rehospitalization at KSBL within 6 months after discharge, *n* (%)	51 (21.8%)	Data of 14 patients missing (died)
Death	18 (7.3%)	--
Death (in hospital death)	14 (5.6%)	--
Death (30 days mortality)	4 (1.6%)	--

* NT-proBNP normal range < 125 ng/L.

** Troponin-T hs < 14 ng/L.

Other variables of interest were body mass index (BMI), a medical history of dyslipidemia, diabetes, or previous PE. Laboratory values of interest were serum N-terminal pro B-Type natriuretic peptide (NT-proBNP) and Troponin-T high-sensitive (hs). The analysis of LOHS was primarily on patients that were discharged alive, a sensitivity analysis was performed on the full data set.

Further, the housing situation before admission and admission *via* another doctor (usually the GP) were entered into the models. For re-hospitalization outcome, we further entered the variable if GP follow-up was suggested and the LOHS.

For descriptive statistics as measures of central tendency, we displayed mean and standard deviation (SD) in case of normal distribution and median with interquartile range in case of skewed distribution, which was assessed through histograms assessment. For categorical variables we reported absolute and relative frequencies.

Variables with missing values were imputed using the k-Nearest Neighbor algorithm [function knn.impute from the R package “bnstruct” (19)]. A zero-truncated negative binomial regression was conducted to estimate the LOHS and its association with potential risk factors using the R package “VGAM.” As a sensitivity analysis, all regression models were additionally performed on the original, non-imputed data set.

Logistic regression models were created to estimate the risk of death and rehospitalization, and its association with potential risk factors.

All statistical analyses were performed using R, version 4.0.3 statistical software (R Foundation for Statistical Computing). All *p*-values reported were 2-sided; statistical significance was defined as *p* < 0.05.

## 3. Results

### 3.1. Patient characteristics

A total of 378 patients were identified who received the diagnosis of pulmonary embolism during their hospital stay. After the exclusion of 24 patients who declined research consent, 15 patients in which the diagnosis was not confirmed with imaging methods, 67 patients who were diagnosed more than 12 h after admission, and a further 24 who had incomplete diagnostic documentation, 248 cases were analyzed. The patient characteristics are shown in Table 1.

The median age at admission was 73 years (range 19–96) and 51.6% were female. The majority of the patients had general insurance (80%). Vital signs measured at admission revealed that the mean HR was 92.1 but one third of the patients presented to the hospital with tachycardia (30.6%). The average blood pressure was 141/83 mmHg, while half of the patients had hypertension while only a minority of the patients had hypotension, (4.4%). Body temperature was usually in the normal range and oxygen saturation was on average 93.7%, but some of the patients needed oxygen supply at entry (14.5%).

The majority of the patients had comorbidities (92.3%), the three most frequent disease types were: cardiovascular (34.7%), mental (22.2%) and chronic lung diseases (17.7%). Patients’ history revealed that previous VTE occurred in 23.4%, the most frequent one was DVT, followed by PE whereas 12 patients had both (4.8%).

Pulmonary embolism severity index (PESI) score was calculated at admission in 19% of the cases, in the remaining 81% of the cases the PESI score was calculated retrospectively. The mean of the PESI score was 96.8 (SD = 31.4). Regarding the laboratory values, NT-proBNP was measured in 71.8% of the patients and the values had a median of 499 ng/l (IQR 125–2,479), whereas troponin-T hs was measured in 62% of the patients with a median of 16.9 ng/l (6.84–46.2).

Before admission most of the patients lived independently, but 14.9% were admitted from a care facility. Almost half of the patients were sent to the hospital by a GP (49.2%). After the discharge, a follow-up with a GP was organized in 29% of all cases. The majority of the patients returned to their private homes (80.6%) while 13.7% were transferred to a care facility or a rehabilitation center (8.5%).

Out of 248 hospitalized patients with pulmonary embolism, 14 patients died during the hospital stay and were excluded from regression analyses with outcome LOHS and re-hospitalization. Patients with PE who did not die within the hospital stayed for a median of 5 days (IQR 3–8). Additionally, four patients died within 30 days (1.6% of the total patients) and rehospitalization at KSBL within 6 months after discharge occurred in 21.8% of the cases.

### 3.2. Prediction of LOHS

Our primary aim was to identify factors that predict LOHS. Table 2 provides coefficient estimates for predictors of LOHS in patients who did not die. Regression coefficients are shown as incident risk ratio (IRR). Patients with higher PESI scores (IRR = 1.068, 95%CI [1.034–1.104], value of *p* < 0.001), higher troponin values [IRR = 1.433, 95%CI (1.189–1.727), value of *p* < 0.001], and with diabetes [IRR = 1.293, 95%CI (1.007–1.66), value of p 0.044] had significantly longer LOHS.

**Table 2:** Results of multivariable zero-truncated negative binomial regression model for length of hospital stay (LOHS) estimation in pulmonary embolism survivors (*n* = 234).

	LOHS prediction	IRR (95%CI)	Pr(>|*z*|)
(Intercept):1	5.899	2.422 (1.742–5.722)	0.004
PESI score (per 10 points)	**6.273**	**1.068 (1.034–1.104)**	**<0.001**
NT**-**proBNP (per 1,000 units)	6.012	1.021 (0.997–1.045)	0.089
Troponin-T hs (per 100 units)	**8.297**	**1.433 (1.189–1.727)**	**<0.001**
Pervious PE	6.485	1.107 (0.829–1.478)	0.492
Previous DVT	5.482	0.924 (0.724–1.178)	0.522
Diabetes	**7.517**	**1.293 (1.007–1.66)**	**0.044**
Cardiovascular diseases	6.904	1.183 (0.969–1.443)	0.098
Dyslipidemia	5.041	0.842 (0.66–1.075)	0.168
BMI	6.039	1.026 (0.87–1.209)	0.763
Housing situation before admission	5.104	0.854 (0.656–1.112)	0.241
Sent by doctor	5.98	1.015 (0.846–1.217)	0.873

Statistically significant variables *p* < 0.05.

The LOHS prediction at the intercept (5.899 days) is the LOHS when all covariates are at 0 (for categorical covariates) or at their mean (for continuous covariates). The predicted LOHS of the model for each variable is presented for one unit increase. If the PESI score increases by one unit (on the original scale per 10 points), the predicted LOHS increases from 5.89 to 6.27 days. A higher increase occurs when the Troponin increases by one unit (on the original scale per 100 n/L) the predicted LOHS rise to 8.3 days. People with diabetes compared to those without tend to stay two nights longer, assuming all other variables are held constant.

Our secondary aims included the analyses of factors associated with mortality and rehospitalization rates. The results of the univariate logistic regression models for mortality, adjusted for PESI score, are displayed in Table 3. Higher PESI scores and NT-proBNP values were significantly associated with mortality in patients with pulmonary embolism [OR 1.617, 95%CI (1.359–1.981), value of *p* < 0.001 and OR 1.091, 95%CI (1.012–1.171) value of *p* 0.013, respectively]. No other variable was found to be statistically significant in the association with the mortality rate.

**Table 3:** Results of univariate logistic regression model for mortality (in-hospital or within 30 days) in pulmonary embolism (*n* = 18).

	OR (95%CI)	Pr(>|*z*|)
PESI score	**1.617 (1.359–1.981)**	**<0.001**
NT-proBNP	**1.091 (1.012–1.171)**	**0.013**
Troponin-T hs	0.139 (0.003–1.949)	0.249
Previous DVT	0.41(0.022–2.322)	0.408
Diabetes	0.782 (0.152–2.979)	0.74
Cardiovascular diseases	1.628 (0.537–5.073)	0.388
Dyslipidemia	0.341 (0.043–1.611)	0.229
BMI	0.443 (0.117–1.399)	0.199
Housing situation before admission (care facility)	1.443 (0.367–4.715)	0.575
Sent by doctor	1.423 (0.454–4.715)	0.548
LOHS	1.056 (0.967–1.137)	0.172

All variables were adjusted for pulmonary embolism severity index (PESI) score. Statistically significant variables *p* < 0.05.

The results of our secondary multivariable analysis concerning rehospitalization rate are reported in Table 4. The odds for rehospitalization within 6 months in KSBL were also significantly higher for patients with a higher PESI score and for patients with a higher LOHS [OR 1.183, 95%CI (1.041–1.353) value of *p* 0.012 and OR 1.099, 95%CI (1.031–1.183) value of *p* 0.007, respectively]. No other variable was found to be statistically significant in the association with rehospitalization.

**Table 4:** Multivariable logistic regression model for rehospitalization in pulmonary embolism (*n* = 51).

	OR (95%CI)	Pr(>|*z*|)
PESI score	**1.183 (1.041–1.353)**	**0.012**
NT-proBNP	0.934 (0.831–1.03)	0.546
Troponin-T hs	0.853 (0.265–1.926)	0.957
Previous PE	0.522 (0.116–1.772)	0.339
Previous DVT	1.346 (0.495–3.456)	0.545
Diabetes	1.917 (0.733–4.893)	0.175
Cardiovascular diseases	1.871 (0.873–4.013)	0.106
Dyslipidemia	0.525 (0.181–1.372)	0.208
BMI	0.747 (0.36–1.437)	0.404
Housing situation before admission (care facility)	0.815 (0.279–2.139)	0.69
Sent by doctor	1.019 (0.501–2.076)	0.959
Follow up with GP	1.352 (0.626–2.869)	0.435
LOHS	**1.099 (1.031–1.183)**	**0.007**

Statistically significant variables *p* < 0.05.

## 4. Discussion

This retrospective observational cohort study of patients with PE showed that LOHS is influenced by PESI score, serum troponin values and diabetes. Other factors such as medical history, other types of comorbidities and whether the patients were sent to the hospital by a GP were not associated with longer LOHS.

Pulmonary embolism severity index score is a validated prognostic model for PE devised by Aujesky and colleagues (20). Originally it was developed to predict 30-day mortality using 11 clinical criteria [age at entry, sex, history of cancer, history of chronic lung disease, history of heart failure, respiratory rate, hypothermia (below 36 degrees Celsius), systolic blood Pressure <100 mmHg, heart rate ≥110 bpm, oxygen saturation below 90%, altered mental status, and respiratory rate ≥ 30 breaths per minute (20)]. The PESI score was also implemented to identify low-risk patients who might be treated outside the hospital and consequently be eligible for early discharge (18). In line with other studies, our results also confirm the prognostic validity of the PESI score in predicting the length of hospital stay (21, 22). The role of PESI in LOHS was confirmed by Rodriguez et al. (23), however the impact of the single items composing the score was unclear. For this reason, in our study, we have also analyzed the items of the PESI score separately and we found that age, sex heart rate over 110 bpm, oxygen saturation <90% and heart failure were significantly predictive for LOHS (for more details see Table A1 in the appendix).

A strong finding of our research is that serum troponin was statistically significant in association with LOHS, despite the model being controlled for cardiovascular disease. This trend was noted by Muktar et al. in 2018 where patients who had long LOHS had higher values of cardiac biomarkers compared to those with short LOHS, but no statistically significant difference was found in their study, possibly due to the small sample size (22). Elevated cardiac troponins are known to indicate subendothelial ischemia in the right ventricle (24) and to be associated with right ventricular dysfunction and pulmonary hypertension in acute PE (25, 26). These complications may explain the found association with extended LOHS.

Another point worth discussing is the fact that diabetes was the only health condition associated with longer LOHS. Previous studies have investigated the relationship between diabetes and PE incidence (27) or demonstrated that patients with diabetes have worse outcomes compared to patients without diabetes, especially in terms of mortality (28) or hospitalization rate (29). A recent study by Schmitt et al. found that PE patients with diabetes had prolonged LOHS (30), which is confirmed by our results. So, despite advances in treatments, diabetes is still associated with a higher risk of adverse outcomes and healthcare providers should take this finding into account. Although PE patients who also suffer from diabetes are at elevated risk for adverse events and a complicated clinical course (30, 31), further studies are required in order to clarify the underlying mechanisms and impact of disturbed glucose metabolism on the generation and clinical outcome of PE in light of LOHS.

The study followed up patients until late 2020 (the first year of the COVID-19 pandemic), however only one case out of 248 in our sample was tested positive for the SARS-CoV-2 virus. The reason why the proportion of patients with a positive test is low relies on our inclusion criteria, since we selected patients who were hospitalized with pulmonary embolism as their main diagnosis (reason for hospitalization). The majority of patients hospitalized with COVID-19 received a different ICD code as their main diagnosis and are not captured in our cohort.

Our secondary aim was to assess which factors were associated with morality rate. The overall in-hospital (5.6%) and the 30-days mortality rate (5.6 and 1.6%) observed in this study, were relatively low compared to that reported in recent studies by Matskiv et al. and by Jiménez et al. (32) (11.9 and 5.4%, respectively). Our results showed that mortality was associated with elevated values of NT-proBNP in addition to the PESI score. As previously stated, the PESI score is the most validated prognostic model for PE in predicting mortality and our study confirms this association and is in line with previous publications (17, 18, 20, 21, 33, 34).

The role of biomarkers in all-cause mortality of patients with pulmonary embolism has been debated. A meta-analysis by Lega et al. (35) showed that higher level of NT-proBNP was associated with higher risk of adverse outcomes, all-cause mortality among them. Interestingly in our research, we detected a difference between the role of NT-proBNP and troponin values. As previously discussed, our study revealed that higher serum troponin was significantly associated with LOHS whereas NT-proBNP was associated with mortality. The meta-analysis of Klok et al. (36) found that high concentrations of BNP in PE patients were associated with complicated in-hospital course and death, while LOHS was analyzed in particular. Another meta-analysis by Beccatini et al. (37) showed that elevated values of troponin were indicator of high risk for short-term death in patients with acute PE, but the study limited his research to troponin values only and did not include NT-proBNP values. In a recent Swiss study by Benmachiche et al. (38) results revealed that patients with high levels of NT-proBNP were at higher risk of in-hospital mortality and longer LOHS, regardless of their clinical characteristics. Although in other conditions like acute coronary syndrome, the level of NT-proBNP provided better predictive power than troponin (39), this difference in PE patients has still to be established. Ultimately, the precise role of biomarkers in early risk stratification is fundamental since PE may present with a wide spectrum of symptoms but in some cases with no evident symptoms. Biomarkers might be fundamental in order to detect a serious condition and allow consequently treatment adjustment with more aggressive therapy.

**Figure 1:**
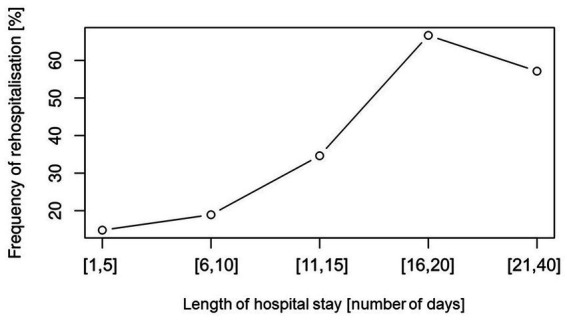
Frequency of rehospitalization versus length of hospital stay (LOHS). Rehospitalization rates increases with longer length of hospital stay in approximately lineal relationship.

Our secondary outcomes included rehospitalization rates. We detected that in our study population rehospitalization within 6 months was significantly influenced by PESI score and in addition by LOHS but not by other factors. PESI score has been validated in studies with a relatively short-term follow-up (30 and 90 days mortality), (17, 33, 40) one study showed its accuracy in predicting long-term prognosis (6 month and 1 year mortality) (33) but its accuracy in predicting long term outcome in terms of rehospitalization in 6 months has not been established. The role of LOHS and its association with the risk of rehospitalization has been debated in research (41–44). Literature suggests that both short length of stay and long length of stay can be associated with rehospitalization rates. On one hand, a shortening of the length of stay could point to the so colloquially called “bloody” discharges, where the patient is not yet in a sufficient state of health or still has open problems (45, 46). On the other hand, a longer length of stay could be associated with rehospitalizations because it could occur especially in critically ill people and multimorbid elderly patients, who are subsequently exposed to a higher risk of readmission (41, 43). In order to test the association of LOHS with rehospitalization we visualized the frequency of rehospitalization versus LOHS and detected rather a linear tendency, as the LOHS increased so did the percentage of rehospitalization rates (Figure 1). For the above-mentioned reasons, we included LOHS in our model as a continuous variable and did not dichotomize it in short versus long length of hospital stay. The explanation for such tendency is that patients with pulmonary embolism hospitalized in KSBL are on average old and multimorbid patients. The positive aspect of these results is that patients were usually not discharged too early and a short length of stay did not result in a higher risk of rehospitalization.

In all our analyses the PESI score had significant influence on LOHS, mortality and rehospitalization. PESI score is an essential parameter for determining how to proceed with the patient (e.g., outpatient treatment, surveillance) (47). Therefore, whenever it is not calculated, the patient is at risk of being under- or overtreated. By definition, the PESI score is used to predict 30 day mortality and is a widely validated model that uses clinical parameters to stratify patients into five risk levels (20).Unfortunately, in our study PESI score was only calculated at the time of admission in 19% of the cases, in the remaining 81% of the cases the PESI score was calculated retrospectively.

Published audits on the management of PE patients only cover very particular aspects of the process, for instance, the use of clinical decision rules (48) or treatment strategies (49) or only look at single subsegmental PE (50) and do not focus on the frequency of the PESI score calculation. Therefore, do not know if the proportion is representative of usual practice. In our opinion, the fact that the PESI score is poorly reported can be due to two factors: either other indicators are given priority in the emergency department, or the PESI was actually calculated but not entered into the patients’ records.

The criteria that make up the score refer to easily accessible information and vital signs that can be measured by a GP. Moreover, the score is of high importance in predicting not only severity and mortality but also LOHS and the likelihood of re-hospitalization. Therefore, we believe that it may be appropriate for GPs who suspect pulmonary embolism to already calculate the PESI when sending a patient to the hospital.

### 4.1. Comparison between multivariable models and sensitivity analysis

**Figure A1:**
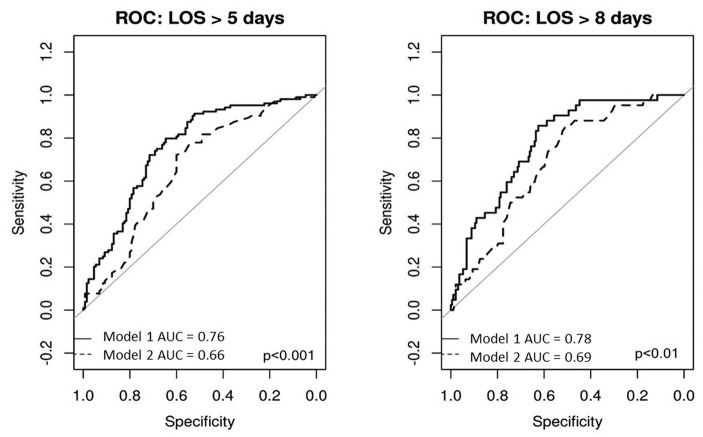
Receiver operating characteristic (ROC) curve accuracy of the models to predict 5 days and 8 days length of hospital stay. For both 5 days and 8 days prediction, model 1 showed higher accuracy than model 2. Model 1 includes the following variables: pulmonary embolism severity index (PESI) score, body mass index (BMI), a medical history of dyslipidemia, diabetes or previous PE, NT-proBNP and troponin-T hs, housing situation before admission and admission *via* GP. Model 2 only includes the PESI score. Pulmonary embolism severity index score is based age at entry, sex, history of cancer, history of chronic lung disease, history of heart failure, respiratory rate, hypothermia [below 36°C], systolic blood pressure (BP) <100 mmHg, heart rate (HR) ≥110 bpm, oxygen saturation below 90%, altered mental status, and respiratory rate ≥ 30 breaths per minute (18).

In our study we assessed the association between PESI score in addition to other risk factors and we also assessed the parameters composing the PESI score alone. As displayed in the Appendix Figure A1 LOHS prediction the receiver operating characteristic (ROC) curve shows a higher accuracy for the model with other parameters in addition to PESI score compared to the model with PESI score alone and this is valid both for 5 days prediction (median LOHS) and for 8 days prediction (the upper IQR).

The sensitivity analysis on the full data for the prediction of LOHS did not give any other predictor except for NT-proBNP which was predictive for in-hospital death. The sensitivity analysis on the full data for the prediction of the PESI items only on LOHS shows the same results as the analysis performed on the patients that were discharged alive.

### 4.2. Strengths, limitations, and further research

Despite the availability of a large amount of data regarding the prognosis of PE patients, only a few studies have investigated possible predictors of LOHS in these patients (15, 21, 51). The novelty of our study is that the analysis did not limit its focus just to the PESI score. Our analysis looked at a broader range of variables (demographic, health-related risk factors and the role of primary care) as well as the PESI score and determined their associations with outcomes of interest. The data collection involving manual extraction of information was conducted by a doctor alone but subsequently, the parameters forming the PESI score were reviewed independently by two researchers. The characteristics of the sample of this study are comparable with other cohorts with a bigger sample size, for example in our study the median age was 73 years 52% were female and 50% had hypertension as a comorbidity. In a German study from 2018 with almost 1 million PE patients, the median age was 72, 54% were female and 43% had hypertension (8).

All statistical analyses that were primarily performed on imputed data have also been applied to the non-imputed dataset, the statistical significance of the variables with missing data did not differ in the two models. The sensitivity analysis demonstrated that the internal validity of our research was not impacted by missing data.

There are some limitations to this study. As a retrospective study design, the data quality depends on precise documentation in the patient files. Particularly we assumed that if the PESI score was not reported, the clinician did not calculate it, so we could have underestimated the percentage of patients with a PESI calculation at entry to hospital. Additionally, information about rehospitalization within 6 months was only possible within KSBL: due to privacy policy, it was not possible to access information about rehospitalizations in other hospitals. However, in Switzerland readmissions usually occur within the same hospital; a Swiss study has shown that only 17% of unplanned readmissions occurred at a different hospital (41). Our results concerning mortality and rehospitalization rates must be interpreted carefully since their occurrence was relatively low (18 and 51 patients, respectively).

Further research to prospectively validate the statistical model’s accuracy in predicting LOHS—ideally in multicenter studies with a larger sample size—is needed.

## 5. Conclusion

Understanding the factors that are associated with LOHS in patients with PE has clinical implications and may help healthcare providers to deliver efficient care and to allocate adequate resources in the management of these patients. In summary, the results of this study showed that the PESI score is a major predictor of LOHS, mortality and rehospitalization in PE patients. Diabetes is an additional risk factor that healthcare providers should be aware of. Even though cardiac biomarkers and comorbidities are predictors of LOHS, their role in defining mortality and rehospitalization is yet to be established. Moreover, our study confirmed the essential role of PESI score calculation in the management of PE patients, clinicians and GPs should be aware of and perform the calculation as soon as PE is diagnosed.

## Data availability statement

The dataset presented in this article is not readily available because it contains sensitive human personal data. All data generated were analyzed during this study and the results included in this article. Further inquiries can be directed to the corresponding author. Requests to access the datasets should be directed to JL, joerg.leuppi@ksbl.ch.

## Ethics statement

The studies involving human participants were reviewed and approved by Nordwest- and Zentralschweiz ethic commission (Project-ID 2021–00964). The ethics committee waived the requirement of written informed consent for participation.

## Author contributions

GL-C and CK were responsible of data collection. GL-C, SG, and MB were responsible for data analysis. GL-C, SG, and JL were responsible for designing the study. All authors contributed to the article and approved the submitted version.

## Funding

The project was financed by the Swiss Personalized Health Network (SPHN Grant # 2018DRI08).

## Conflict of interest

The authors declare that the research was conducted in the absence of any commercial or financial relationships that could be construed as a potential conflict of interest.

## Publisher’s note

All claims expressed in this article are solely those of the authors and do not necessarily represent those of their affiliated organizations, or those of the publisher, the editors and the reviewers. Any product that may be evaluated in this article, or claim that may be made by its manufacturer, is not guaranteed or endorsed by the publisher.

## Appendix

**Table A1:** Multivariable zero-truncated negative binomial regression model for length of hospital stay (LOHS) estimation in pulmonary embolism survivors with pulmonary embolism severity index (PESI) items separately assessed (*n* = 234).

	LOHS prediction	IRR (95%CI)	Pr(>|*z*|)
Intercept	4.638	1.774(1.148–2.742)	0.01
Age	**5.207**	**1.138 (1.071–1.21)**	**<0.001**
Sex	**5.864**	**1.296 (1.083–1.55)**	**0.005**
History or active cancer	4.72	1.02 (0.829–1.255)	0.851
BP systolic < 100 mmHg	5.452	1.197 (0.761–1.883)	0.436
Heart rate ≥ 110 bpm	**6.139**	**1.361 (1.075–1.724)**	**0.01**
Respiratory rate ≥ 30 breaths/min	4.876	1.058 (0.707–1.583)	0.783
Oxygen < 90%	**6.188**	**1.373 (1.109–1.699)**	**0.004**
Chronic lung disease	5.107	1.114 (0.878–1.414)	0.375
Altered mental status	5.003	1.089 (0.51–2.324)	0.826
Hypothermia < 36°C	4.821	1.045 (0.612–1.782)	0.872
Heart failure	**9.345**	**2.113 (1.165–3.832)**	**0.014**

The analysis of the items composing the PESI score showed that age, sex, heart rate over 110 bpm, oxygen saturation <9% and heart failure were significantly associated with LOHS. Statistically significant variables *p* < 0.05.

## Abbreviations

BMI: Body mass index
BP: Blood pressure
BPM: Beats per minute
CI: Confidence interval
CTPA: Computed tomography pulmonary angiogram
DVT: Deep vein thrombosis
GP: General practitioner
HR: Heart rate
ICD: International Classification of Diseases
IQR: Interquartile range
IRR: Incident risk ratio
KSBL: (Kantonsspital Baselland) cantonal hospital of Baselland
LOHS: Length of hospital stay
NT-proBNP: N-terminal pro b-type natriuretic peptide
OR: Odds ratio
PE: Pulmonary embolism
PESI: Pulmonary embolism severity index
ROC: Receiver operating characteristic
SD: Standard deviation
VTE: Venous thromboembolism
